# The PI3K/AKT Pathway—The Potential Key Mechanisms of Traditional Chinese Medicine for Stroke

**DOI:** 10.3389/fmed.2022.900809

**Published:** 2022-05-31

**Authors:** Chenyang Gu, Qiankun Zhang, Yajing Li, Rong Li, Jia Feng, Wanghao Chen, Waqas Ahmed, Ismatullah Soufiany, Shiying Huang, Jun Long, Lukui Chen

**Affiliations:** ^1^Department of Neurosurgery, Neuroscience Center, Integrated Hospital of Traditional Chinese Medicine, Southern Medical University, Guangzhou, China; ^2^Department of Cardiology, Laboratory of Heart Center, Zhujiang Hospital, Southern Medical University, Guangzhou, China; ^3^Department of Neurosurgery, Shanghai 9th People Hospital Affiliated to Shanghai Jiaotong University School of Medicine, Shanghai, China; ^4^School of Medicine, Southeast University, Nanjing, China; ^5^School of Traditional Chinese Medicine, Southern Medical University, Guangzhou, China

**Keywords:** Traditional Chinese Medicine, stroke, herb, PI3K/Akt pathway, neuroprotection

## Abstract

Stroke is associated with a high disability and fatality rate, and adversely affects the quality of life of patients and their families. Traditional Chinese Medicine (TCM) has been used effectively in the treatment of stroke for more than 2000 years in China and surrounding countries and regions, and over the years, this field has gleaned extensive clinical treatment experience. The Phosphatidylinositol 3 kinase (PI3K)/protein kinase B (AKT) pathway is important for regulation of cell migration, proliferation, differentiation, and apoptosis, and plays a vital role in vascularization and oxidative stress in stroke. Current Western medicine treatment protocols for stroke include mainly pharmacologic or mechanical thrombectomy to restore blood flow. This review collates recent advances in the past 5 years in the TCM treatment of stroke involving the PI3K/AKT pathway. TCM treatment significantly reduces neuronal damage, inhibits cell apoptosis, and delays progression of stroke via various PI3K/AKT-mediated downstream pathways. In the future, TCM can provide new perspectives and directions for exploring the key factors, and effective activators or inhibitors that affect occurrence and progression of stroke, thereby facilitating treatment.

## Graphical Abstract

The schematic diagram of neuroprotection of TCM herbs for stroke *via* the PI3K/AKT-mediated pathways and key downstream molecules. 
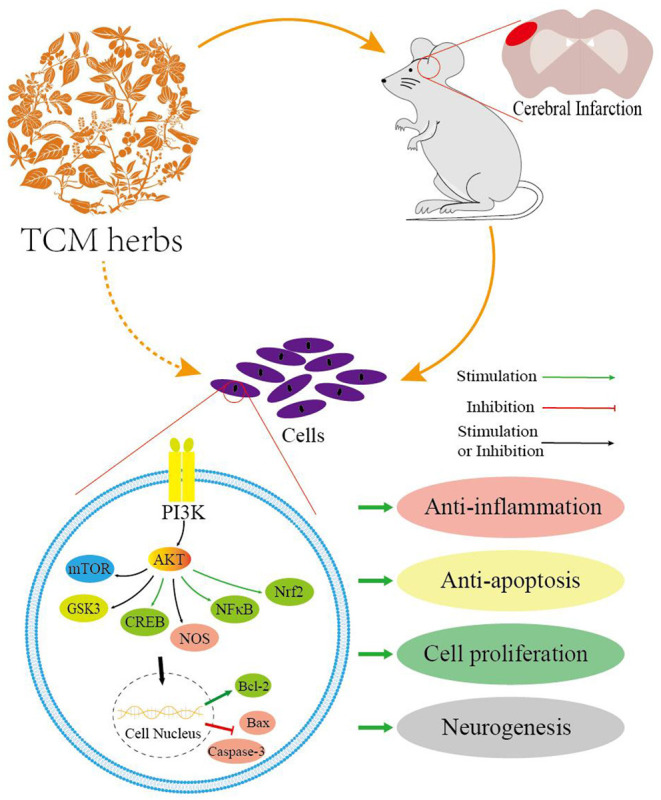

## Introducation

Stroke, called “Zhongfeng” in Chinese, is still a major disease that seriously endangers human life and health, and is associated with a high rate of disability and fatality (1). Globally, about 80 million patients are afflicted with stroke, and the number of patients in China has reached more than 14 million. Among them, about 1.8 million patients die every year (2, 3). Most of the remaining survivors have varying degrees of disability, which seriously affects the quality of life of patients and their families; stroke takes a toll on the body, spirit, emotions of patients, and also causes economic loss. From the perspective of pathogenesis, 85% of strokes are ischemic strokes, which are caused by intracranial vascular occlusion, leading to apoptosis or necrosis of cells in the brain, and changes in neurovascular units and brain damage. Secondary cell death after ischemic stroke is coordinately mediated by multiple pathophysiological mechanisms, such as inflammation, oxidative stress, vascular dysfunction, energy depletion, excitotoxicity, autophagy, and apoptosis (4, 5).

Current treatment strategies for ischemic stroke mainly include mechanical thrombectomy or intravenous thrombolysis with recombinant tissue-type plasminogen activator to restore blood flow; however, the narrow time window limits the effectiveness and the safety of treatment (6); hence, finding other more effective treatments is imperative.

There is an urgent need for developing new medicines for stroke; new types of chemical compounds or drugs in modern medicine, requiring extensive chemical analysis and animal experiments for confirmation of their therapeutic effect. Based on the various ancient Chinese medicine texts and treatises, there are many prescriptions for treatment of stroke in Traditional Chinese Medicine (TCM), including decoctions, powders, injections, oral solutions, as well as acupuncture and scraping. When applied to animal models, some Chinese medicines reduce cerebral infarction, inhibit autophagy and cell apoptosis, and improve the long-term neurological recovery after stroke.

Phosphotylinosital 3 kinase (PI3K)/Protein kinase B (AKT) is an important pathway that regulates cell migration, proliferation, differentiation, and apoptosis, and plays a vital role in physiological or pathophysiological conditions. PI3K is a family of proteins that can catalyze the G-phosphate transfer of ATP to the phosphatidylinositol of D3 position (7); After activation, the signal sent by AKT can be transmitted to diverse substrates with various functions. Abnormal activity or upregulation or downregulation of the pathway may cause a variety of diseases, such as neurodegenerative diseases, stroke, diabetes, cancer, etc. (8). The activation of PI3K may be mediated by tyrosine kinase growth factor receptors. PI3K transforms phosphatidylinositol 4,5-bisphosphate into phosphatidylinositol 3,4,5-triphosphate, combines AKT and Pyruvate Dehydrogenase Kinase Isozyme 1 (PDK1), and causes PDK1 to phosphorylate AKT, which is the direct target downstream protein. The activation of the PI3K/AKT pathway causes a cascade reaction of downstream proteins that mediate cell function. In various organs, the repair is basically carried out through PI3K/AKT pathway, especially central nervous system.

## Mechanisms of PI3K/AKT Pathway in Stroke

### Vascularization

The PI3K/AKT pathway has several downstream target receptors, for example, c-kit receptor, insulin-like growth factor-1 receptor, vascular endothelial growth factor (VEGF) receptor, etc. Angiogenesis is one of the downstream effects. There are two main routes. One is the PI3K/AKT/nitric oxide synthase (NOS) pathway, which promotes the release of nitric oxide (NO) by vascular parietal cells. NO is released from the endothelium, causing vasodilation by the activity of endothelial NOS (eNOS) (9). NO, fibroblast growth factors (FGFs), VEGFs, along with others, stimulate the generation of new blood vessels. Hypoxia upregulates hypoxia-inducible factor 1α (HIF-1α) levels; epidermal growth factor receptor (EGFR) and PI3K also stimulate HIF-1α production. The other pathway is the PI3K/AKT/mammalian target of rapamycin (mTOR) pathway that induces the release of HIF-1α (10). What is puzzling is that it induces the protein HIF-1α, but the mRNA level is normal. Perhaps the PI3K/AKT/mTOR pathway might enhance HIF-1α translation. This pathway is useful in the current context. Examples include: the synergy of anti-PD-1 and endostar on PI3K/AKT/mTOR-mediated autophagy (11); ginsenoside Rg1 promotes cerebral vascularization *via* the PI3K/AKT/mTOR pathway after stroke (12).

### Oxidative Stress

To deal with oxidative damage, the body is equipped with an efficient defense system that detoxifies and eliminates harmful chemicals and inactivates reactive oxygen species (ROS). Over past the 20 years, people have sought to examine the link between the PI3K/AKT pathway and oxidative stress. Nuclear factor-erythroid 2-related factor 2 (Nrf2), a main regulator of various antioxidant enzymes, is the primary downstream target of the PI3K/AKT pathway. Nrf2 modulates the balance of cell redox and senses the status of oxidative stress by the activity of antioxidant factors, such as ferritin, glutathione reductase, thioredoxin reductase, glutathione peroxidase, superoxide dismutase (SOD), hemeoxygenase-1 (HO-1), and NAD(P)H: quinone oxidoreductase 1 (NQO1) (13, 14). The consensus of some studies on Nrf2 phosphorylation is that the PI3K/AKT/Nrf2 pathway may perhaps be the main pathway that withstands oxidative stress to cells (15, 16). This could be an effective therapeutic target for aging, which has been shown to be linked to oxidative stress.

## Stroke in TCM

According to the *Yellow Emperor's Classic of Medicine—Suwen* (17), the main mechanisms of stroke are related to internal injuries and accumulation, excessive emotion and desire, improper diet, and fat body. Internal injuries and accumulation: as a person's age increase, *Yang* gradually becomes self-deficient, and *Yang* turns to *Yin*. Imbalance of work and rest, prolonged diseases, and *Yin* causes damage to the *qi* of the organs. The deficiency of *qi* causes inability to supply blood and cerebral meridian stasis. Excessive emotions and desires: anger injures the liver, cause *qi* stagnation in the liver. This *qi* stagnation transforms liver heat, causing disorder of *qi* and blood of the liver. The disorder invades the brain, causing blood overflow or blocks the cerebral meridian. Improper diet: the spleen (pancreas), stomach, and liver are usually in harmony. High-fat and high-alcohol diet can hurt this harmony and cause endogenous phlegm fever. The phlegm fever and blood stasis block each other, and heat accumulation leads to cerebral meridian stasis. Fat body: there is deficiency of *qi* and phlegm-damp develops in fat bodies, which causes the stagnation of *qi* and blood. Both these also invade the brain, causing cerebral meridian stasis. From the perspective of TCM, therapeutic strategies include clearing the phlegm fever to sputum, draining turbidity of organs, replenishing *qi* and strengthening the body, removing blood stasis, and promoting blood circulation, nourishing the liver and its harmony. The prescription that is used commonly is the Buyang Huanwu Decoction (BHD), which mainly includes *Salvia miltiorrhiza, Scutellaria baicalensis* Georgi, *Ligusticum wallichii, Astragalus propinquus* Schischkin.

## Active Extracts of Chinese Medicines

Chinese herbal medicines afford therapeutic effects. Many neuroprotective drugs have been widely proven to be effective in animal experiments, but they are not effective when applied to patients. When applied to animal stroke models, several Chinese herbal medicines reduce cerebral infarction, inhibit autophagy and cell apoptosis, improve long-term neurological recovery through similar or identical pathways; the downstream substrates mainly include mTOR, glycogen synthase kinase 3 (GSK3), nuclear factor kappa light chain enhancer of activated B cells (NF-κB), Nrf2, NOS, cyclic AMP-responsive element binding protein (CREB), etc.

### PI3K/AKT/mTOR

As a downstream substrate that transmits signals after AKT activation, mTOR, a serine threonine kinase, mediates cell growth, survival, and metabolism (18).

**Figure 1:**
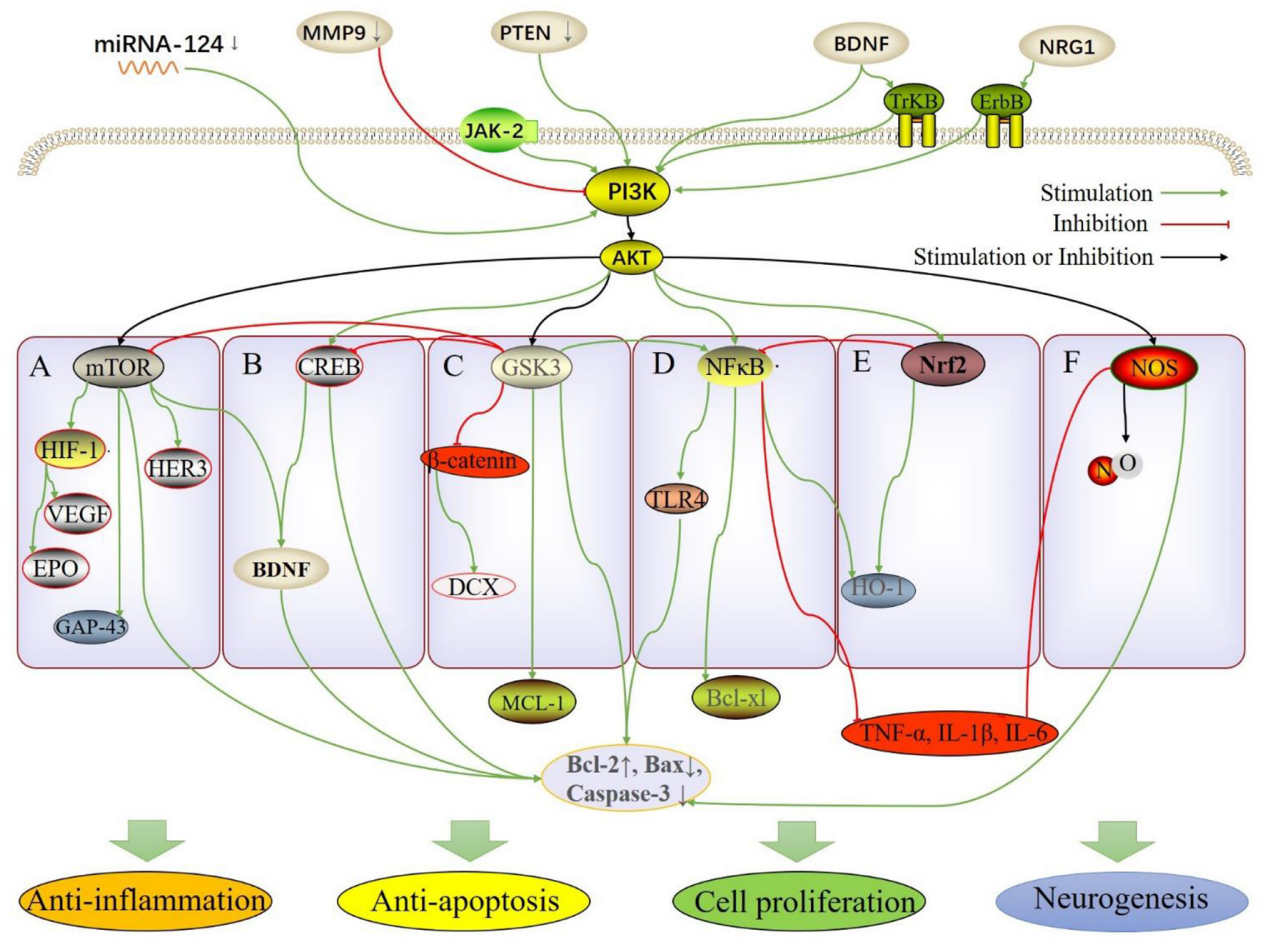
The molecular and pathway mechanisms involved in the neuroprotective effect of TCM herbs, including anti-inflammation, anti-apoptosis, cell proliferation and neurogenesis. **(A)** PI3K/AKT/mTOR; **(B)** PI3K/AKT/CREB; **(C)** PI3K/AKT/GSK3; **(D)** PI3K/AKT/NF-κB; **(E)** PI3K/AKT/Nrf2; **(F)** PI3K/AKT/NOS.

**Figure 2:**
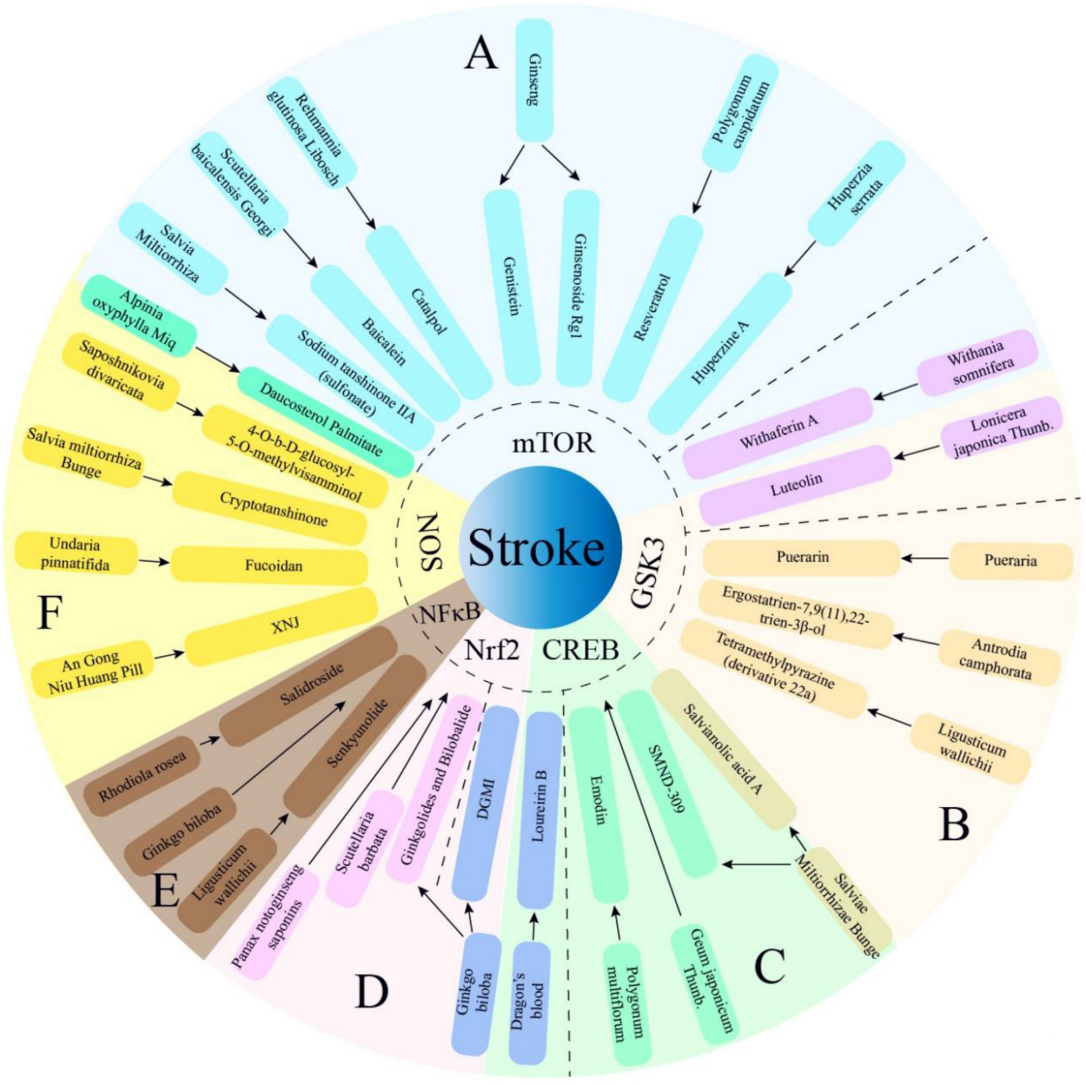
Multiple active components from TCM herbs exert therapeutic effects via PI3K/AKT -mediated 6 main pathways. The TCM herbs of manuscript not mentioned above almost exert therapeutic effects via PI3K/AKT -mediated Bcl-2 pathway. SMND-309, a chemical derivant of propenoic acid of Sal A; DGMI, Diterpene ginkgolides meglumine injection; XNJ, Xingnaojing. **(A)** PI3K/AKT/mTOR; **(B)** PI3K/AKT/GSK3; **(C)** PI3K/AKT/CREB; **(D)** PI3K/AKT/Nrf2; **(E)** PI3K/AKT/NF-κB; **(F)** PI3K/AKT/NOS.

**Tanshinone IIA**, a natural component derived from Salvia miltiorrhiza (Dan-Shen in Chinese), has been proven to have therapeutic effects in cerebrovascular and cardiovascular diseases. Tanshinone IIA treatment promotes the recovery of brain function and increases neuronal viability; furthermore, the glucose concentration in the serum and cultured medium was increased, possibly regulated by an increased glucose uptake ability and activation of the PI3K/AKT/mTOR pathway (19). Sodium tanshinone IIA sulfonate is a water-soluble derivative of tanshinone IIA, which suppresses the expression of HIF-1 and hemoglobin genes *via* inhibition of the PI3K/AKT/mTOR pathway, thereby protecting against hypoxia in cerebral hemorrhage in zebrafish (20). **Catalpol**, an iridoid glycoside compound, the main active component of Rehmannia glutinosa Libosch (Di-Huang in Chinese), is commonly used in TCM for treating diseases of aging and stroke (21). Catalpol activates PI3K/AKT/mTOR/neuromodulin and PI3K/AKT/mTOR/brain-derived neurotrophic factor (BDNF) pathways with or without inhibition of miR-124 to further improve neuronal survival, cell viability, and axonal growth after oxygen and glucose deprivation/reoxygenation (OGD/R) (22, 23). Baicalein (5,6,7-trihydroxyflavone), the main active component extracted from the root of Scutellaria baicalensis Georgi (Huang-Qin), has shown promise when administered in the acute phase of ischemic stroke (24). Baicalein significantly inhibited autophagy *via* activation of the PI3K/AKT/mTOR pathway and subsequently induced changes to B cell lymphoma-2 (Bcl-2), caspase-3, and Bcl-Associated X (Bax) proteins (25). **Daucosterol palmitate**, the main active component of Alpinia oxyphylla Miq. fruit (Yi-Zhi in Chinese), exhibits therapeutic effects in diarrhea, cognitive impairment, intestinal disorders, and diuresis (26). Daucosterol palmitate protects neurons and inhibits neuronal apoptosis by activating the PI3K/AKT/mTOR pathway-mediated β-actin (27). **Cornus officinalis**, Shan-Zhu-Yu in Chinese, clinically used in liver and kidney deficiency, has been combined with other herbs to treat stroke. Cornel iridoid glycoside is the main active component extracted from Cornus officinalis; this therapy exerted neuroprotective effects in middle cerebral artery occlusion (MCAO) in rats during acute and chronic phases, thereby improving motor functions and promoting recovery of somatosensory deficits, and alleviated memory deficits. The effects were partially due to promotion of angiogenesis and neurogenesis of gray matter, and anti-neuroinflammatory and anti-apoptotic effects *via* increased expression of BDNF and neuregulin-1V proteins and consequently activation of the PI3K/pAKT/pmTOR pathway (28). **Genistein**, a natural phytoestrogen extracted from soybeans, was found to have neuroprotective potential and could help in preventing stroke. Genistein therapy could alleviate neuronal apoptosis induced by I/R injury in ovariectomized rats *via* activation of the PI3K/AKT/mTOR pathway (29). **Ginsenoside Rg1**, one of the most active components of ginsenoside that is extracted from ginseng (Ren-Shen in Chinese), is used widely for the treatment of various neurological diseases (30), mediates cell proliferation and differentiation, inhibits inflammation, cell apoptosis, and has other pharmacological effects (31). Ginsenoside Rg1 promotes migration, proliferation, vascularization, and tube formation in endothelial cells through increased expression of VEGF and HIF-1 *via* activation of the PI3K/AKT/mTOR pathway after ischemic stroke (12). **Resveratrol** (3,4,5-trihydroxystilbene) is a phenolic ingredient found in Polygonum cuspidatum (Hu-Zhang in Chinese) and also exists abundantly in red grape skin and red wine. Resveratrol improves neurological function and alleviates neurological damage in cerebral infarction, inhibits apoptosis, and protects hippocampal neurons from damage induced by I/R injury *via* activation of the Janus kinase 2 (JAK2)/PI3K/AKT/mTOR pathway (32). **Huperzine A**, a lycopodium alkaloid derived from Huperzia serrata (Qian-Ceng-Ta in Chinese), is commonly used for treating Alzheimer's disease in clinical practice because of its inhibitory effect on acetylcholinesterase. Huperzine A reduces oxidative glutamate toxicity in hippocampal cells and inhibits apoptosis *via* activation of the BDNF/PI3K/AKT/mTOR pathway and the consequent increase in Bcl-2 and decrease in caspase-3 (33). **Withaferin A** is a steroidal lactone extracted from Withania somnifera (Ashwagandha, Zui-Qie in Chinese) (34), which exhibits immunomodulatory, anti-inflammatory, cardioprotective, and neuroprotective effects (35). Withaferin A exhibits neuroprotective effects, including anti-apoptotic effects and promotes cell proliferation by suppressing phosphatase and tensin homolog deleted on chromosome ten (PTEN) and subsequent activation of the PI3K/AKT/mTOR and PI3K/AKT/GSK3β pathways, and inhibition of migration of vascular smooth muscle cells (36). The molecular mechanism and effect of TCM active components in this section through the PI3K/AKT/mTOR pathway is briefly summarized in Figures 1A, 2A.

### PI3K/AKT/GSK3

GSK3 is a ubiquitous serine-threonine kinase with various cellular and neurophysiological functions, including neurogenesis, neurotrophicity, neuroprotection, mood stabilization, and anti-inflammation (37, 38).

As mentioned above, **Withaferin A**, from Withania somnifera, exhibits neuroprotection by suppressing PTEN and subsequently activating the PI3K/AKT/mTOR and PI3K/AKT/GSK3β pathways, among which the GSK3 pathway is also a key pathway for neuroprotection in TCM (36). Similarly, Luteolin, 3′,4′,5,7-tetrahydroxyflavone, is a flavonoid, a natural compound, present widely in fruits and vegetables (39). **Luteolin** enhances cell viability and inhibits apoptosis by downregulating the matrix metalloproteinase 9 expression and activating the PI3K/AKT-mediated mTOR and GSK3β pathways, which alleviate cerebral infarction (40). **Tetramethylpyrazine** (TMP) is an active component of Ligusticum chuanxiong (Chuan-Xiong in Chinese), which is commonly used in TCM clinically for the treatment of cardio- and cerebrovascular diseases (41). TMP functionally increases transcription of redox proteins, scavenges free radicals, and protects antioxidant enzymes (42). Besides, TMP very effectively penetrates the blood–brain barrier (BBB). A compound called α-tetramethylpyrazinyl-N-tert-butyl nitrone (TBN), a TMP nitrone conjugate, shows potent free radical scavenging ability and crosses the BBB easily, protecting neurons from ischemic stroke (43). **Caffeic acid**, a hydroxyl extractive of cinnamic acid possessing a phenolic moiety (44), and its ester derivatives show excellent neuroprotection (45). The chemical combination of TBN and caffeic acid is compound 22a, which maintains mitochondrial membrane potential and inhibits cell apoptosis by activating the PI3K/AKT/GSK3β pathway, thereby protecting against glutamate-induced cerebellar granule neuronal damage (46, 47). The compound **7,9(11),22-ergostatrien-3β-ol** (EK-100) is the main active component extracted from the ethyl acetate crude extract of Antrodia Camphorate (Aphyllophorales, Niu-Zhang-Zhi in Chinese) (EtOAc-AC), a well-known TCM (48). EtOAc-AC and EK100 reduce apoptosis and inflammation by decreasing NF-κB and caspase 3, and promoting Bcl-2 and double-cortin (DCX) by activating the PI3K/AKT pathway, inhibiting GSK3, and activating β-catenin (49). **Puerarin** is a phytoestrogen extracted from Pueraria (Ge-Gen in Chinese). Puerarin affords protection in various diseases, such as cardiac dysfunction, liver injury, and neurological diseases (50); it is especially neuroprotective in Parkinson's disease (51), Alzheimer's disease (52), and ischemic stroke (53). Puerarin improves cell viability in the hippocampus significantly, inhibits cell apoptosis, and protects the hippocampus by activating the PI3K/AKT/GSK-3β pathway and consequently increase in myeloid cell leukemia 1, and inhibition of caspase-3 (54). **Salvianolic acid A** (Sal A) is another main active component of Salvia miltiorrhiza that is similar to Tanshinone IIA. Studies suggest that SalA has potent protective effects against ischemia-induced injury both *in vitro* and *in vivo* (55). Sal A attenuates inflammation/nitrosative stress and preserves the integrity of the BBB by inhibiting NF-κB. Sal A concomitantly promotes neurogenesis by activating the PI3K/AKT pathway to induce GSK3 to enhance catenin/DCX and Bcl-2 expression (56). Sal A involves CREB when it has anti-inflammatory effects *via* the GSK3-mediated activated Bcl-2 pathway. The molecular mechanism and effect of TCM active components in this section through the PI3K/AKT/GSK3 pathway is briefly showed in Figures 1C, 2B.

### PI3K/AKT/CREB

CREB, a nuclear transcription factor, can be stimulated by phosphorylated AKT, which facilitates CREB-binding protein translocation to the promoter and triggers the formation of protective proteins (57).

Similarly, a compound obtained from Salvia miltiorrhiza, **SMND-309**, a chemical derivative of propenoic acid of Sal A (58), is a novel metabolite produced in the brains and hearts after oral administration of Sal A, which elicits strong neuroprotective effects by promoting angiogenesis and inhibiting cell apoptosis and cerebral edema. SMND-309 prevents apoptosis of differentiated SH-SY5Y cells by increasing cell viability and reducing lactate dehydrogenase (LDH) activity after OGD/R by activating the PI3K/AKT/CREB pathway (59). Geum japonicum Tunb. var. chinense (Lan-Bu-Zheng in Chinese) has been commonly used in the treatment of dizziness and headaches in China. As the main active components, **Tellimagrandin II**, 5-desgalloylstachyurin, and 3,4,5-Trihydrox-ybenzaldehyde have multiple therapeutic effects, including anti-inflammation (60), anti-apoptosis (61), and so on. The extracts protect astrocytes from OGD/R-induced injury by inhibiting apoptosis and astrocyte reactivity, *via* activation of the BDNF/PI3K/AKT/CREB pathway (62). **Emodin**, an anthraquinone derivative derived from Polygonum multiflorum Thunb. (He-Shou-Wu in Chinese), is an effective apoptotic agent promoting oxidative damage at high concentrations (63). Use of emodin appears counterintuitive due to its toxicity; however, it also affords protection to multiple organs (64). Natural emodin exerts neuroprotective effects and is used in the treatment of neurodegenerative disease (65). Emodin inhibits glutamate-induced apoptosis and has significant neuroprotective effects *via* activation of the PI3K/AKT-mediated Bcl-2 and CREB/BDNF pathways, and has been shown to enhance behavioral function in I/R injury (66). **Dragon's blood** (DB, Long-Xue in Chinese) is a rare and precious traditional medicine in China. It is abundant in four different plant genera: Dracaena, Croton, Daemonorops, and Pterocarpus (67). DB has the properties of anti-inflammation and anti-oxidative stress (68). **Loureirin B**, the phenolic extract of DB, is beneficial in patients diagnosed with ischemic stroke clinically (69). Meantime, Longxuetongluo capsule (the main ingredient is Loureirin B) was approved by the Chinese government as a new drug for treating ischemic stroke (70). Loureirin B activates the PI3K/AKT/Nrf2 and CREB pathways, which increase HO-1 and Bcl-2 expression to protect cells from OGD/R injury. Ginkgo biloba, Yin-Xing in Chinese, has various therapeutic effects, including “dispersing toxin” (clearance of inflammation and oxidative state), treatment of stroke and myocardial infarction; this has been recorded in the “Compendium of Materia Medica, Bencao Gangmu in Chinese,” written with Li Shizhen in the Ming Dynasty (71). **Ginkgolides**, the major component of Diterpene ginkgolides meglumine injection derived from Ginkgo biloba, inhibits neuronal apoptosis for protection against cell injury induced by OGD/R by activation of the PI3K/AKT-mediated CREB and Nrf2 pathways (57). The molecular mechanism and effect of TCM active components in this section through the PI3K/AKT/CREB pathway is briefly concluded in Figures 1B, 2C.

### PI3K/AKT/Nrf2

Nrf2, a transcription factor mediating endogenous anti-oxidants, plays a key role in intracellular defense against ROS (72). Furthermore, the activation of Nrf2 exerts protective effects on BBB integrity after brain injury (73).

As mentioned above, extract of Ginkgo biloba, including bilobalide and ginkgolides, belong to ginkgolide terpenoid lactones. **Bilobalide** and **ginkgolides** upregulate the levels of antioxidant proteins, including HO-1, SOD, and NQO1 *via* activation of the important PI3K/AKT/Nrf2 pathway to protect neurons against oxidative stress. **Scutellaria barbata D. Don** (SBD), which belongs to the Scutellaria genus (Ban-Zhi-Lian in Chinese), is a perennial herb in China with therapeutic effects, including anti-oxidation and anti-inflammation (74). SBD treatment promotes cell proliferation and viability, reduces cell apoptosis after OGD/R injury, and improves mitochondrial dysfunction and oxidative injury *via* activation of the PI3K/AKT-dependent Nrf2 pathway (75). **Panax notoginseng saponins** (PNS) are the main active components of Xue-Sai-Tong Injection, commonly used in the treatment of cardiac diseases and ischemic stroke in China (76), which protect neurons against I/R injury (77), and inhibit efficiently oxidant activity combined with above ginsenosides (12, 78). As an extrinsic regulator, Panax notoginseng saponins protect against OGD/R-induced BBB disruption *via* activation of the PI3K/AKT/Nrf2/HO-1 pathway (79). **Salidroside** is a key bioactive component of Rhodiola rosea (Hong-Jing-Tian in Chinese), which has neuroprotective effects and improves cognitive functions (80). Salidroside reduces cerebral infarction and neurological deficits in MCAO; it reduces neuroinflammation and neural damage *via* activation of the PI3K/AKT/Nrf2/NF-κB pathway (81). Salidroside exerts its neuroprotective effects through the PI3K/AKT pathway that involves another key factor, NF-κB. The molecular mechanism and effect of TCM active components in this section through the PI3K/AKT/Nrf2 pathway is generalized in Figures 1E, 2D.

### PI3K/AKT/NF-κB

NF-κB is a key transcription factor that, respectively, regulate cellular responses to inflammation (82).

Identically, it has been reported that **terpenoids** and **flavonoids** in Ginkgo biloba possess potent anti-inflammation, anti-oxidative, and free radical scavenging activity (83). Ginkgetin aglycone treatment reduces oxidative stress and inflammation to protect against neuronal injury in stroke *via* activation of the PI3K/AKT/NF-κB/TLR4/Bcl-2 pathway (84). Moreover, **Senkyunolide-H** (SEH) is another main bioactive component of Ligusticum wallichii (85). SEH inhibits inflammatory factor release and exerts anti-apoptotic effects *via* activation of the PI3K/AKT/ NF-κB pathway and has therapeutic potential in ischemic stroke (86). **Senkyunolide-I**, the stereoisomer of SHE, exhibits definite anti-apoptotic activity in I/R injury and has anti-inflammatory effects against endotoxin insult (87). The molecular mechanism and effect of TCM active components in this section through the PI3K/AKT/ NF-κB pathway is briefly concluded in Figures 1D, 2E.

### PI3K/AKT/NOS

Nitric oxide, synthesized from L-arginine, plays a crucial role in controlling blood pressure, blood flow, and oxygen delivery (88). The NOS isoforms include inducible NOS (iNOS), endothelial NOS (eNOS) and neuronal NOS (nNOS) (89).

Extracted from Salvia miltiorrhiza identically, **cryptotanshinone**, another main active component, possesses various pharmacological activities, including anti-cancer (90), anti-inflammation (91), anti-oxidative effects (92). Cryptotanshinone exhibits anti-apoptotic and blood vessel protective activities and is neuroprotective against cerebral stroke *via* inhibition of the PI3K/AKT/eNOS pathway and subsequent increased Bcl-2 and NO in both the cerebral cortex and the peripheral blood (93). As a nutritious food in East Asia, **Undaria pinnatifida**, Qun-Dai-Cai in Chinese, is traditionally used in soup recipes to promote breast milk production during dyslipidemia and the postnatal period (94). Fucoidan, extracted from Undaria pinnatifida, contains sulfate and L-fucose, and possesses bioactivities, including anti-inflammation, anti-coagulation, anti-tumor, and neuroprotection (95). **Xingnaojing** (XNJ) is extracted from An Gong Niu Huang Pill and is used for treating stroke; it contains She-Xiang (Moschus), Jiang-Huang (Curcuma longa L.), Bing-Pian (Borneol), and Zhi-Zi (Gardenia), and is approved by the Chinese Government (96). XNJ reverses brain injury, promotes functional recovery, and exerts neuroprotective effects after stroke (97). XNJ inhibits human brain microvascular endothelial cell (HBMEC) apoptosis after I/R injury and OGD injury *via* activation of the PI3K/AKT/eNOS pathway and consequently causes increase in Bcl-2/Bax and decrease in caspase-3 (98). **40-O-b-D-glucosyl-5-O-methylvisamminol** (OGOMV), derived from Saposhnikovia divaricate (Fang-Feng in Chinese), is commonly used in the treatment of cancer, inflammation, and cardiac events in China (99). OGOMV attenuates cleaved caspase-3a,−9a related apoptosis, and inflammation to exert neuroprotection *via* inhibition of the P13K/AKT/iNOS pathway (100). The molecular mechanism and effect of TCM active components in this section through the PI3K/AKT/NOS pathway is briefly introduced in Figures 1F, 2F.

### Others

There are many active components of TCMs that directly increase Bcl-2 and decrease Bax *via* activating PI3K/AKT pathway to exert neuroprotective effects.

**Ligustilide**, derived from Ligustilide walliichi, attenuates cerebral infarction, and reverses neurological injury, thereby affording neuroprotection, reverses apoptosis of hippocampal cells *via* activation of the PI3K/AKT pathway and consequently increases Bcl-2, and decreases Cyt C, Bax, and caspase-3 (101). **Tetramethylpyrazine** protects Bone Mesenchymal Stem Cells (BMSC) against H2O2-induced apoptosis *via* activation of the PI3K/AKT pathway; this was used to improve cell survival in combination with BMSCs in ischemic stroke (102). Tetramethylpyrazine leads to neural progenitor cell (NPC) migration and survival by inducing stromal cell derived factor 1 secretion, stimulating the PI3K/AKT pathway (103). **Chrysophanol** (CHR), the main active component of Rhubarb (Da-Huang in Chinese), promotes cell viability and inhibits cell apoptosis by increasing miR-216a expression and activating the PI3K/AKT pathway (104). Kaempferol-3-O-rhamnoside, an active ingredient extracted from Schima wallichii Korth (Hong-Mu-He in Chinese) leaves, reduces cerebral infarction and water content, and inhibits apoptosis by activating the PI3K/AKT pathway (105). **Astilbin**, a dihydroflavonol derivative from Rhizoma Smilacis glabrae (Tu-Fu-Ling in Chinese), significantly improves cerebral infarction and neurological deficits, and inhibits apoptosis and inflammation after I/R injury by activating the PI3K/AKT pathway (106). **Icariin** is a quinlizidine flavone extracted from Epimedium L. (Yin-Yang-Huo in Chinese); this in combination with MSCs greatly improves cerebral infarction, neurologic deficits of motor and somatosensory function, and promotes angiogenesis and neurogenesis *via* activation of PI3K/AKT pathway and consequently increases VEGF and BDNF (107).

Besides these, there are several TCMs and their active ingredients that exert neuroprotection *via* different PI3K/AKT-mediated pathways.

**Salidroside**, derived from Rhodiola rosea, activates the PI3K/AKT pathway to induce production of HIF-α subunits and erythropoietin, one or more of which mediate anti-inflammatory effects after IR injury (108). **Baicalin**, a flavonoid compound derived from Scutellaria baicalensis root, effectively inhibits apoptosis, and reduces cerebral infarction and neuron loss *via* activation of the PI3K/AKT-mediated glutamate transporter 1 (109). **Vinpocetine** is an alkaloid derivative isolated from the leaves of Phyllostachys pubescens (Mao-Zhu in Chinese), which possesses anti-inflammation and anti-platelet aggregation properties, and improves cognition, cerebral blood flow, and metabolism (110). Vinpocetine reduces cerebral infarction and edema in I/R injury, and reduces apoptosis, inflammation, and oxidative stress induced by I/R injury *via* activation of the PI3K/AKT-mediated connexin43 phosphorylation pathway (111). **Artesunate** is a water-soluble derivative of artemisinin, found in the aboveground dry part of Artemisia annua L (Qing-Hao in Chinese). Artesunate enhances NSC proliferation in the infarcted cortex and alleviates I/R injury *via* inhibition of the Forkhead box protein O 3a transcription by inducing phosphorylation and downregulating p27 through the PI3K/AKT pathway (112). **Formononetin** is one of the main components of astragalus isoflavones, derived from Astragalus membranaceus (Huang-Qi), widely used for neuroprotection and neurological functional recovery (113). Formononetin helps in recovery of injured nerve functions, and mediates therapeutic effects through neuronal differentiation and synaptic plasticity after ischemic stroke *via* activation of the PI3K/AKT/ERK (extracellular regulated protein kinases) pathway (114). **DL-3-n-butylphthalide** is an oily component extracted from Apium graveolens Linn (Chinese celery), which attenuates neurovascular damage (115), improves cognitive functions and neurological outcomes (116), and reduces brain edema (117), inflammation and oxidation (118), and cerebral infarction. DL-3-n-butylphthalide activates primary and secondary dendrites and dendritic tips *via* inactivation of the PI3K/AKT pathway, without involving the above mentioned up/downstream pathways (119).

## Composite Decoction

Reports suggest that composite Chinese medicines have multiple pharmacological interactions. There are many prescriptions for stroke treatment in TCM, including decoctions, powders, injections, oral solutions, in combination with acupuncture and scraping.

**BHD** is the most representative TCM formula for stroke treatment, first documented in Yilin Gaicuo of Wang Qingren in the Qing Dynasty; it includes Astragalus membranaceus, Dang-Gui (Angelica sinensis), Ligusticum chuanxiong, Shao-Yao (Paeonialactiflora), Hong-Hua (Carthamustinctorius), Tao-Ren (seed of Prunus persica), and Di-Long (Lumbricus). BHD attenuates neurological deficits, promotes proliferation (120), neurorehabilitation (121) and the migration of NPCs to ischemic areas (122), and induces synaptic plasticity (121). BHD significantly improves the proliferation and differentiation of NSCs, cerebral infarction, and neuron viability, and decreases cell apoptosis *via* activation of the PI3K/AKT/Bad and Jak2/Stat3/Cyclin D1 pathways (123). **Hou's Black Pulvis** (Houshi Heisan, HBP), a classic compound prescription, was first documented in “Synopsis of Golden Chamber (Jingui Yaolue)” by Zhang Zhongjing in the Han Dynasty (216 A.D.); it is widely used for recovery of neuronal function in China, and mainly includes Ju-Hua (chrysanthemum), Bai-Zhu (Atractylodes macrocephala Koidz), Fang-Feng (Saposhnikovia divaricate), Jie-Geng (Platycodon grandifloras). HBP inhibits amyloid precursor protein deposition (124) and reduces the inflammatory reaction (125) by multiple mechanisms, including blunting of abnormal activation of astrocytes and activation of the endogenous nerve growth factor. HBP treatment enhances the reorganization and regeneration of cerebral blood circuits through the BDNF/PI3K/AKT pathway, which also overcomes the growth-inhibitory signals, RhoA/ROCK and Nogo-A/NgR to facilitate plasticity after stroke (126). **Tongnao Decoction** (TND) is a Chinese decoction approved and used in the treatment of ischemic stroke in Jiangsu Province Hospital of TCM; it is composed of Jiu-Jie-Chang-Pu (Rhizoma Anemones altaicae), Tian-Nan-Xing (Rhizoma arisaematis), Ligusticum chuanxiong, Gou-Teng (Ramulus Uncariae Cum Uncis), Tian-Ma (Rhizoma Gastrodiae), Jiang-Can (Bombyx batryticatus), Shui-Zhi (Hirudo), and Hong-Jing-Tian (Radix et Rhizoma Rhodiolae Crenulatae). TND promotes the migration, proliferation, and tube formation of cells, and restores neurovascular function by promoting angiogenesis in the ischemic cerebral microvasculature, *via* activation of the PI3K/AKT and Raf/MEK/ERK pathways (127). **Taohong Siwu Decoction** (TSD) comes from the “Golden Mirror of Medicine” of the Qing Dynasty. It consists of six TCM herbs: seed of Prunus persica, Carthamustinctorius, Angelica sinensis, Ligusticum chuanxiong, Rehmannia glutinosa Libosch and Paeonialactiflora. TSD has significant effects in treating the rats with I/R, the mechanism of which may be involved in promoting the angiogenesis and recovering the nerve function by activating PI3K/AKT signal pathway (128). **Nao-shu-ning** (NSN) is approved by Zhang Jianfu of Shaanxi University of Chinese Medicine, composed of Leonurus artemisia, Radix Cyathulae, Hirudo, Radix Notoginseng, Rhizoma Imperatae, Raw Rhubarb, Fructus Forsythiae and Rhizoma Acori Tatarinowii. The possible mechanism of effect of NSN on nerve regeneration is the inhibition of AKT and inhibiting phosphorylation of Raf-1 during reperfusion (129). In clinical study, **Chaigui Wendan Dingzhi decoction** (CWDD) is composed of 16 TCM herbs, mainly includin*g*, Bupleurum chinense (Chai-Hu), Paeonialactiflora, Poria cocos, Pinellia ternate (Ban-Xia) and seed of Ziziphus jujuba Mill. CWDD has a significant effect on the recovery of cognitive function and depressive mood in stroke patients after stroke which may be related to the activation of PI3K/AKT-mediated Bcl and BAX pathway (130).

## Conclusion and Prospect

Stroke has one of the highest mortality rates globally. Currently used medicines and modern medical surgical treatment are not conducive to recovery. This review collates knowledge and experience on stroke management from the realm of TCM with modern molecular scientific research and describes the progress TCM involving PI3K/AKT pathway regarding neuroprotection in the past 5 years. The PI3K/AKT pathway is the key pathway that underpins pathophysiological mechanisms of diseases, also stroke, effects including neurological damage, cell apoptosis, vascularization, oxidative stress, etc. The PI3K/AKT pathway acts as a central mediator of growth factor stimulation during cell growth (131). Previous studies have shown that the PI3K/AKT inhibited Raf/MAPK/ERK pathway-mediated negative effects during cerebral I/R (132). They suggest that the PI3K/AKT pathway contributes to cyto-protection in different cell, tissue, and animal models, including focal ischemia and post-ischemic conditioning. TCM treatment involving the PI3K/AKT pathway has considerable effects in reducing neuronal damage, inhibiting cell apoptosis, and delaying the progression of stroke.

The neuroprotection of herbal single components through the PI3K/AKT pathway is detailed in the main text, some herbs contain multiple active components that synergistically protect the nervous system through PI3K/AKT-mediated downstream molecules. There are four active components of Salvia miltiorrhiza, including Sodium tanshinone IIA sulfonate, Sal A, SMND-309, and Cryptotanshinone, exert neuroprotection synergistically through the four PI3K/AKT-mediated pathways, including increasing neuronal viability, preventing apoptosis and promoting neurogenesis (20, 56, 59). Ginkgo biloba has various therapeutic effects, and contains multiple active components, like Ginkgolides, bilobalide, Ginkgetin aglycone. They inhibit neuronal apoptosis, reduce oxidative stress and inflammation *via* activation of the PI3K/AKT pathways (57, 84). Compound 22a and Senkyunolide-H, are derived from Ligusticum wallichii. Both maintains mitochondrial membrane potential, inhibits cell apoptosis and inflammatory factor release by activating the PI3K/AKT pathways (46, 47, 86). Extracted from Scutellaria baicalensis Georgi, Baicalein and Baicalin inhibits autophagy, reduces cerebral infarction and neuronal loss *via* activation of the PI3K/AKT mediated pathway (25, 109).

In the past, the prevention and treatment of stroke was often guided by vascular recanalization, including thrombolysis, mechanical recanalization, etc. This review shows more than 20 common TCM herbs for stroke, each of which has at least one active ingredient that can improve the progression of stroke through PI3K pathway mediated downstream pathways. Some components may also interact with each other in positive or negative feedback during the treatment process to achieve dynamic balance by mutual regulation. Like *Salvia miltiorrhiza, Ginkgo biloba, Scutellaria baicalensis* Georgi, etc., multiple active components of a single herb can synergistically activate the PI3K/AKT pathway to exert a variety of neuroprotective effects, including maintaining neuronal vitality, neurogenesis, preventing apoptosis, reducing inflammation, and oxidative stress, etc. The compound formula is composed of a variety of traditional Chinese medicines, and different herbs can also play a synergistic effect to form a multi-target and multi-organ protective effect on the body. Perhaps the PI3K/AKT pathway is a potential key pathway of TCM treatment for stroke, which provides targets and thought for exogenous prevention and treatment. Active ingredients of TCM provides a direction for finding key mechanisms and pathways in other diseases (Graphical Abstract). However, the advantages of TCM for stroke are not absolute. The composition of TCM herbs is complex, and it is impossible for TCM herbs to exert effect only through the PI3K/AKT pathway. It is necessary to further explore the specific multi-target and multi-pathway mechanisms of TCM herbs, which cannot be used blindly. The incompatibility of TCM should be avoided, this may cause discomfort in the digestive system, skin mucous membranes, nervous system and cardiovascular system at light level, and may lead to poisoning with dose-effect in severe level.

The global library of TCM and TCM ingredients is huge, and the mechanism research on TCM ingredients is still lacking. We hope that more researchers will devote themselves to TCM research in the future. Everything starts with nature and ends with nature.

## Author Contributions

CG and QZ collected the data, wrote the manuscript, and conceived the structure of Figures. YL collected the data of manuscript and finished the contents of Figures. RL, JF, and WC summarized and analyzed the data. WA and IS revised the manuscript. LC, JL, and SH launched the viewpoint of manuscript. All authors declared that they materially participated in the article preparation. All authors contributed to the article and approved the submitted version.

## Funding

This study was supported by National Natural Science Foundation of China (81671819) and Department of Education of Guangdong Province (2021ZDZX2011).

## Conflict of Interest

The authors declare that the research was conducted in the absence of any commercial or financial relationships that could be construed as a potential conflict of interest.

## Publisher's Note

All claims expressed in this article are solely those of the authors and do not necessarily represent those of their affiliated organizations, or those of the publisher, the editors and the reviewers. Any product that may be evaluated in this article, or claim that may be made by its manufacturer, is not guaranteed or endorsed by the publisher.

## Abbreviations

AKT: protein kinase B
Bax: Bcl-Associated X
BBB: blood-brain barrier
Bcl-2: B cell lymphoma-2
BDNF: brain-derived neurotrophic factor
BHD: Buyang Huanwu Decoction
BMSC: Bone Mesenchymal Stem Cells
CHR: Chrysophanol
CREB: cyclic AMP-responsive element binding protein
DCX: double-cortin
EA: electroacupuncture
EGFR: epidermal growth factor receptor
eNOS: endothelial NOS
EtOAc-AC: ethyl acetate crude extract of Antrodia Camphorate
FGFs: fibroblast growth factors
GSK3: glycogen synthase kinase 3
HBMEC: human brain microvascular endothelial cell
Houshiheisan, HBP: Hou's Black Pulvis
HIF-1α: hypoxia-inducible factor 1α
HO-1: hemeoxygenase-1
iNOS: inducible NOS
I/R: ischemia-reperfusion
JAK2: Janus kinase 2
LC3: micro-tubule-associated protein 1 light chain 3 II/I
LDH: lactate dehydrogenase
MCAO: middle cerebral artery occlusion
mTOR: mammalian target of rapamycin
NF-κB: nuclear factor kappa light chain enhancer of activated B cells
nNOS: neuronal NOS
NOS: nitric oxide synthase
NQO1: NAD(P)H: quinone oxidoreductase 1
NPC: neural progenitor cell
Nrf2: Nuclear factor-erythroid 2-related factor 2
OGD/R: oxygen and glucose deprivation/reoxygenation
OGOMV: 40-O-b-D-glucosyl-5-O-methylvisamminol
PDK1: Pyruvate Dehydrogenase Kinase Isozyme 1
PI3K: Phosphatidylinositol 3 kinase
PNS: Panax notoginseng saponins
PTEN: phosphatase and tensin homolog deleted on chromosome ten
ROS: reactive oxygen species
SBD: Scutellaria barbata D. Don
SHE: Senkyunolide-H
SOD: superoxide dismutase
TBN: α-tetramethylpyrazinyl-N-tert-butyl nitrone
TMP: Tetramethylpyrazine
TND: Tongnao Decoction
TREM2: microglial triggering receptor expressed on myeloid cells 2
ULK1: Unc-51-like kinase 1
VEGF: vascular endothelial growth factor
XNJ: Xingnaojing.
